# Molecular Dynamics Simulation and Kinetic Study of Fluoride Binding to V21C/V66C Myoglobin with a Cytoglobin-like Disulfide Bond

**DOI:** 10.3390/ijms21072512

**Published:** 2020-04-04

**Authors:** Lu-Lu Yin, Jia-Kun Xu, Xiao-Juan Wang, Shu-Qin Gao, Ying-Wu Lin

**Affiliations:** 1 School of Chemistry and Chemical Engineering, University of South China, Hengyang 421001, China; yinlulu1625@163.com (L.-L.Y.); wxj0207@126.com (X.-J.W.); 2 Key Lab of Sustainable Development of Polar Fisheries, Ministry of Agriculture and Rural Affairs, Yellow Sea Fisheries Research Institute, Chinese Academy of Fishery Sciences, Lab for Marine Drugs and By products of Pilot National Lab for Marine Science and Technology, Qingdao 266071, China; xujk@ysfri.ac.cn; 3 Lab of Protein Structure and Function, University of South China, Hengyang 421001, China; gaoshuqin99@163.com

**Keywords:** heme proteins, protein design, disulfide bond, MD simulation, fluoride ion

## Abstract

Protein design is able to create artificial proteins with advanced functions, and computer simulation plays a key role in guiding the rational design. In the absence of structural evidence for cytoglobin (Cgb) with an intramolecular disulfide bond, we recently designed a de novo disulfide bond in myoglobin (Mb) based on structural alignment (i.e., V21C/V66C Mb double mutant). To provide deep insight into the regulation role of the Cys21-Cys66 disulfide bond, we herein perform molecular dynamics (MD) simulation of the fluoride–protein complex by using a fluoride ion as a probe, which reveals detailed interactions of the fluoride ion in the heme distal pocket, involving both the distal His64 and water molecules. Moreover, we determined the kinetic parameters of fluoride binding to the double mutant. The results agree with the MD simulation and show that the formation of the Cys21-Cys66 disulfide bond facilitates both fluoride binding to and dissociating from the heme iron. Therefore, the combination of theoretical and experimental studies provides valuable information for understanding the structure and function of heme proteins, as regulated by a disulfide bond. This study is thus able to guide the rational design of artificial proteins with tunable functions in the future.

## 1. Introduction

Protein design is able to not only reveal the structure-function relationship of native proteins, but also create artificial proteins with advanced functions [1,2,3,4,5,6,7,8,9,10,11,12]. This is especially the case for heme protein design, which has received much attention in the last few decades, and various approaches have been established for rational design, such as the introduction of non-heme metal ions and unnatural amino acids, and the use of heme mimics to act as an active site [1,2,3,4,5,6,7,8,9,10,11,12]. Importantly, computer modeling and molecular dynamics (MD) simulation play key roles in guiding the protein design [13,14,15,16,17,18]. For example, computer simulation was successfully applied to design a non-heme iron binding site in the heme pocket of myoglobin (Mb), which converted an O_2_ carrier into a functional nitric oxide reductase [19]. With the help of Rosetta matcher, Lu and co-workers rationally designed a [4Fe-4S] cluster in cytochrome *c* peroxidase (C*c*P) that closely mimicked the active site and reproduced the function of native sulfite reductase (SiR) [20].

**Figure 1:**
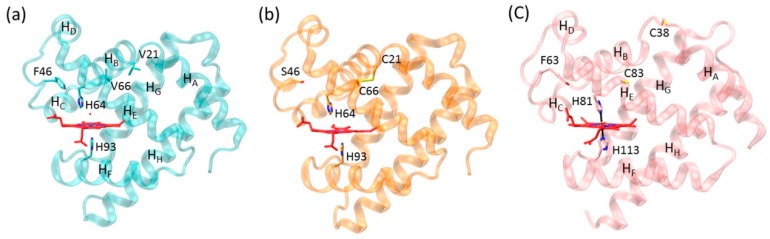
X-ray structure of (**a**) sperm whale Mb (PDB code 1JP6 [31]), (**b**) F46S/V21C/V66C Mb mutant (PDB code 5ZEO [33]), and (**c**) human Cgb (PDB code 1V5H [27]) in their ferric forms. The proximal and distal His residues, the engineered disulfide bond of C21-C66, the reduced residues of C38 and C83, and the eight α-helices (H_A_-H_H_) of Mb and Cgb are highlighted.

Disulfide bond is a common post-translational modification (PTM) of Cys residues, which play crucial roles in regulating the structure and function of proteins [21,22]. For example, manganese peroxidase (MnP) contains five disulfide bonds, and engineering an additional disulfide bond can further increase its tolerance for heat inactivation [23,24]. Disulfide bond has also been shown to regulate the binding of ligands in native heme proteins, as observed for neuroglobin (Ngb) [25,26] and cytoglobin (Cgb) [27,28,29,30]. Note that both Ngb and Cgb are relatively newly discovered globins, with a bis-His coordinated heme and an intramolecular disulfide bond [26,27]. This is different from the O_2_ carrier Mb, which has a H_2_O/His coordinated heme in ferric state without a disulfide bond (Figure 1a) [31], although these proteins belong to the same globin family. Based on their amino acid sequences and structural alignments, we engineered an intramolecular disulfide bond in Mb, F46C/M55C Mb [32], and V21C/V66C Mb [33], which closely mimics that in Ngb (Cys46–Cys55) and Cgb (Cys38–Cys83), respectively. Moreover, we solved an X-ray structure of a triple mutant F46S/V21C/V66C Mb (Figure 1b) [33], which confirms the formation of a de novo designed disulfide bond of Cys21–Cys66.

Although the X-ray crystal structure of human Ngb with an intramolecular disulfide bond was solved previously [26], there is still no 3D structure available for the oxidized form of human Cgb with an intramolecular disulfide bond. Instead, the structure of the reduced form shows that the two sulfur atoms of Cys38 and Cys83 are separated from each other by ~12.3 Å (Figure 1c) [27]. Therefore, as a complement, the X–ray crystal structure of F46S/V21C/V66C Mb provides valuable information for oxidized Cgb. In a recent study, we confirmed that the formation of an intramolecular disulfide bond enhances the protein stability and fine-tunes protein functions such as nitrite reductase and dehaloperoxidase activities [33].

To further probe the structural-functional consequences of Mb with a de novo designed disulfide bond of Cys21–Cys66, we herein used a fluoride ion as a probe, as previously applied for various heme proteins [34,35,36,37,38,39], by performing MD simulation of the fluoride–protein complex. Moreover, to confirm the observation in MD simulation, we determined the kinetic parameters of fluoride binding to the protein. The combination of both theoretical and experimental studies provides valuable information for understanding the structure and function of heme proteins, as regulated by a disulfide bond.

## 2. Results and Discussion

### 2.1. Protein Motions of Fluoride-Mb Complexes

**Figure 2:**
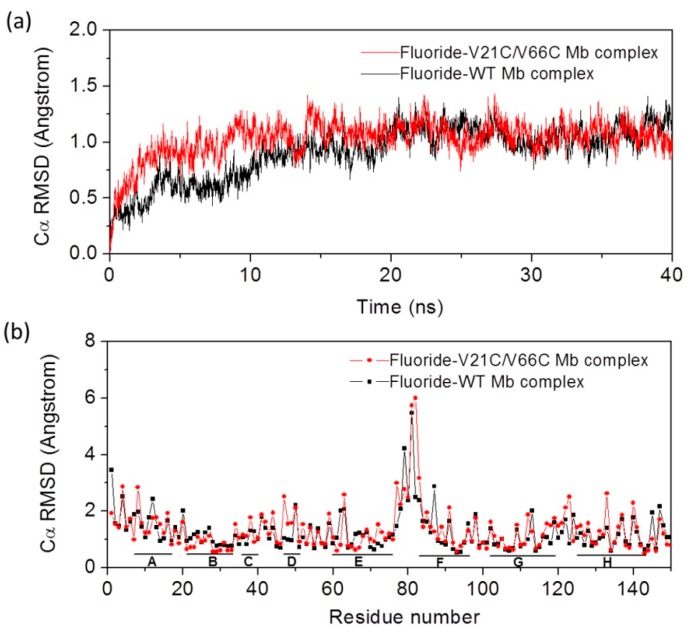
(**a**) Root mean square deviation (RMSD) for fluoride binding to WT Mb and V21C/V66C Mb from the starting structure as a function of time; (**b**) the average RMSD of each residue for the last 20 ns. The eight α-helices (H_A_–H_H_) are indicated by short solid lines at the bottom.

It has been shown that the heme axial water molecule in Mb (Figure 1a) can be removed upon binding of small exogenous ligands such as a fluoride ion, in turn, the fluoride ion can be used as a probe to study the hydrogen (H)-bond interactions in the heme distal site [34,35,36,37,38,39]. To provide dynamics information for V21C/V66C Mb in complex with the fluoride ion, as well as for the fluoride–Wild-type (WT) Mb complex, we performed MD simulations for both protein complexes and monitored the Cα root mean square deviation (RMSD) from the starting structure during 40 ns of MD simulations. As shown in Figure 2a, the RMSD profile of fluoride–V21C/V66C Mb (red) and fluoride–WT Mb (black) complexes fluctuates around 1 Å after ~10 ns and ~20 ns, respectively. This observation indicates that the fluoride–V21C/V66C Mb complex first achieves a stable equilibration of the overall structure compared with that of the fluoride–Mb complex, likely due to the stabilization effect of Cys21-Cys66 disulfide bond.

To provide more detailed information for protein motions, we analyzed the average RMSD over time of each residue for the last 20 ns (Figure 2b). The results show that the eight α-helices in both protein complexes exhibit small deviations around 1 Å, with large deviations observed for the loop regions, especially for the loop of H_E_–H_F_ (residues 75–81), which agrees well with previous MD studies of Mb [40]. It was interesting to observe that most residues in the H_B_ helix where Cys21 is located exhibit small deviations compared to those in WT Mb, and similar situations were observed for most residues in H_E_ where Cys66 is located. These observations suggest that the formation of Cys21-Cys66 disulfide bond enhances the protein stability for the local region, without alteration of the overall protein motions.

### 2.2. Structural Comparison of Fluoride–Mb Complexes

**Figure 3:**
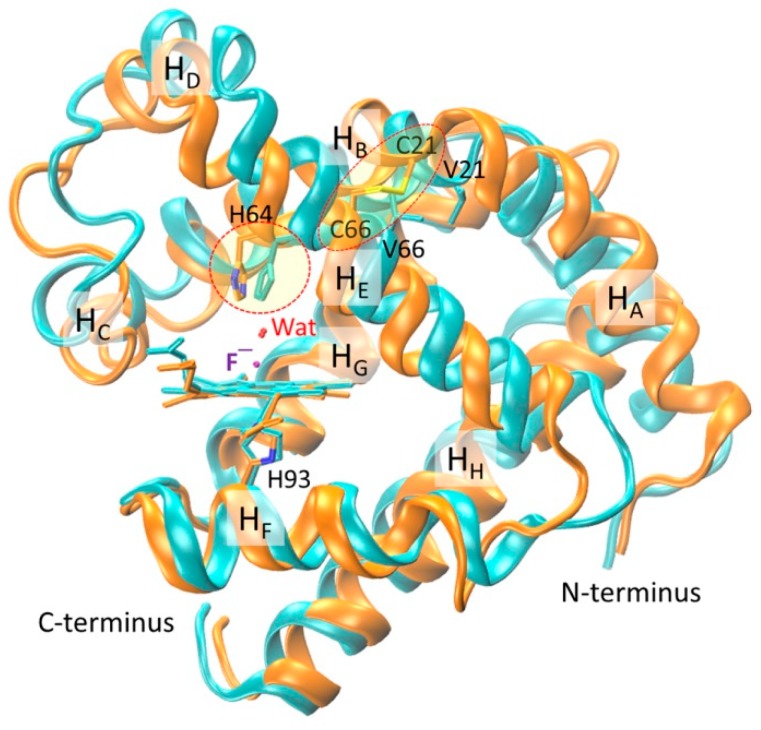
Overlap of modeling structures of fluoride–WT Mb (cyan) and fluoride–V21C/V66C Mb (orange) complexes. The heme group, proximal His93, axial fluoride ion, distal water, N- and C-terminus, and eight α-helices (H_A_–H_H_) are highlighted for clarifications. The conformation of distal His64 and the disulfide bond of C21–C66 are indicated by dashed circles.

With energy minimization and equilibration, the simulated overall structure of the two fluoride–Mb complexes is similar. To make a detailed structural comparison, we overlapped the two structures according to the heme iron center. As shown in Figure 3, the spatial orientations of α-helices H_A_–H_E_ are slightly different between these two complexes, whereas those of the last three α-helices H_F_–H_H_ are very similar to each other. With the formation of Cys21-Cys66 disulfide bond, the loop between H_A_ and H_B_ where Cys21 is located shifted toward the middle of H_E_ where Cys66 is located, which induced a locally structural rearrangement. As a result, both H_C_ and H_D_, including the long loop connecting, underwent a structural rearrangement. Note that the introduction of a de novo disulfide of Cys46–Cys55 in this region mimicking that in native human Ngb regulates the protein structure and enhances the peroxidase activity [32]. These results also agree with the structural comparison between WT Mb and F46S/V21C/V66C Mb triple mutant in their X-ray crystal structures [33], albeit with more protein motions in MD simulations due to in the solution state.

Moreover, as a consequence of structural rearrangement, the spatial orientation of heme distal His64 is different in the two protein complexes (indicated by a circle line in Figure 3), although the heme axial fluoride ions, as well as the distal water molecules, overlapped very well. This observation suggests that the formation of an intramolecular disulfide bond can regulate the conformation of heme distal histidine, thereby fine-tuning the ligand binding property and protein reactivity, as those observed for native Cgb with a bis-His heme coordination [27,28,29,30].

### 2.3. Interactions of Fluoride ion with Mbs

**Figure 4:**
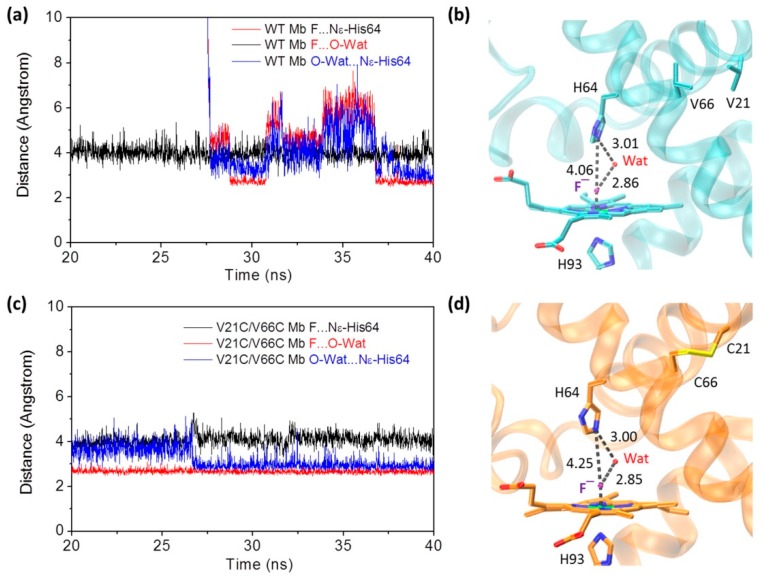
Time-dependence of distances between fluoride ion and the distal His64, fluoride ion and distal water, and distal water and His64, for WT Mb (**a**) and V21C/V66C Mb (**c**), respectively. The modeling structures of WT Mb (**b**) and V21C/V66C Mb (**d**) at the end of simulation. The H-bond interactions in the heme distal site are indicated by dotted lines, as well as the distance (Å).

To provide detailed information for the heme active site of the fluoride–Mb complexes, we monitored the distance between the heme axial fluoride ion and the distal His64 (Nε atom) during the last 20 ns in MD simulation. Moreover, the heme distal water molecule in the equilibrated structure at the end of simulation was also monitored for the last 20 ns. As shown in Figure 4a for the fluoride–WT Mb complex, upon fluoride binding to the heme iron, the distance between the fluoride ion and the Nε atom of distal His64 was ~4.1 Å for the last 20 ns. Note that the distance of Fe-F coordination bond was found to be ~1.8 Å, the same value as optimized in a previous study by Smulevich and co-workers [37]. The heme distal water molecule in the equilibrated structure (Figure 4b) acted as a bridge by forming two H-bonds with a fluoride ion and the distal His64, simultaneously. Moreover, it was interesting to observe that the distal water molecule underwent large distance changes in simulation. By inspecting the MD trajectory, we noted that this was attributed to the existence of several water molecules that may also form H-bond interactions with the fluoride ion and stabilize its coordination. This phenomenon was also observed for fluoride binding to *Thermobifida fusca* hemoglobin in previous MD simulation [37].

In case of the fluoride–V21C/V66C Mb complex (Figure 4c,d), it was found that the heme distal His64 has a slightly large distance to the axial fluoride ion (~4.3 Å). Similarly, a distal water molecule interacted with both the distal His64 and the fluoride ion, by forming an H-bonding network. Although several water molecules were also observed in the MD trajectory, the H-bond distances of the distal water did not change dramatically, especially for the last 12 ns. Note that multiple water molecules were also observed in the X-ray structure of Mb with a modified heme center, such as in L29E Mb mutant containing three water molecules, which was found to regulate ligand binding including the fluoride ion, as well as protein reactivity such as H_2_O_2_ activation [41]. These observations suggest that with the formation of the Cys21–Cys66 disulfide bond and structural rearrangement, the fluoride ion may exhibit a different binding property to the heme iron compared to that binding to WT Mb.

### 2.4. Kinetic Binding of Fluoride ion to Mbs

**Figure 5:**
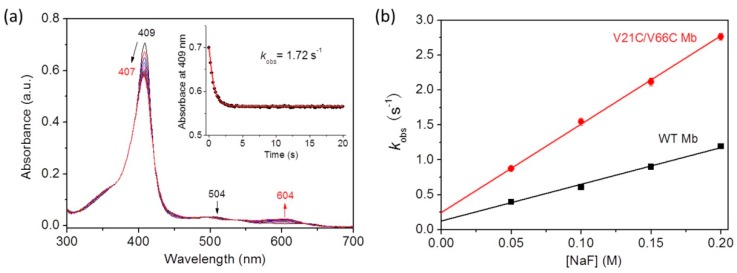
(**a**) Stopped-flow spectra upon mixing V21C/V66C Mb and fluoride (5 μM and 100 mM, respectively, final concentration) in 100 mM KH_2_PO_4_ buffer (pH 7.0) for 20 s. (**b**) Plots of observed rate constants versus fluoride concentrations, with WT Mb shown for comparison.

With the theoretical information from MD simulation, we were also interested in determining the kinetic parameters for fluoride binding to the protein using the experimental method. For this purpose, we performed stopped-flow UV–Vis spectroscopic studies for fluoride binding to V21C/V66C Mb, with WT Mb as a control. As shown in Figure 5a, the spectra showed that the Soret band rapidly shifted from 409 nm to 407 nm upon mixing V21C/V66C Mb with the fluoride ion, with a decrease of 504 nm in absorption and concomitant increase of a charge-transfer (CT1) band at 604 nm, similar to that observed for fluoride binding to a dehaloperoxidase (DHP) (605 nm) [42]. The resultant new spectrum (407 nm and 604 nm) resembles that of the fluoride–WT Mb complex (406 nm and 607 nm), as reported in a previous study [38]. Moreover, as shown in Figure 5a (inset), the absorbance change of the Soret band was fitted well to a single exponential decay equation, indicating the rapid formation of a stable fluoride–protein complex within 5 s.

To obtain the kinetic parameters, we analyzed the results by plotting the rate constants (*k*_obs_) versus fluoride concentrations (Figure 5b). The obtained association rate constant, *k*_a_ (*k*_on_), and the dissociation rate constant, *k*_d_ (*k*_off_), are summarized in Table 1. Note that the *k*_on_ and *k*_off_ values determined for WT Mb are similar to those reported for horse Mb (6.0 ± 0.5 M^−1^s^−1^ and 0.10 ± 0.01 s^−1^) under similar conditions [36]. The results showed that V21C/V66C Mb exhibited a fluoride binding rate constant (*k*_on_) ~2.4-fold higher than that of WT Mb. At the same time, it exhibited a doubly increased dissociation rate constant (*k*_on_). As a result, V21C/V66C Mb showed a slightly enhanced equilibrium for fluoride binding (*k*_on_/*k*_off_) compared to that of WT Mb (Table 1). These results suggest that the conformational change of distal His64 upon formation of the Cys21–Cys66 disulfide bond leads to rapid fluoride binding, as well as rapid dissociation from the heme iron, presumably due to the weakened interactions with distal His64 and water molecules. These results also indicate that the de novo designed disulfide bond of Cys21–Cys66 in Mb plays a fine-tuning role resembling that in Cgb, where Cys38–Cys83 was shown to play a key role in tuning the binding affinity of exogenous ligands by changing the oxidation/reduction state of the disulfide bond [43,44].

## 3. Conclusions

In summary, we performed MD simulation of fluoride–V21C/V66C Mb complex with a de novo designed disulfide bond of Cys21-Cys66 mimicking that in human Cgb, with the fluoride–WT Mb complex as a control. The results show that the formation of Cys21-Cys66 disulfide bond in Mb enhances the protein stability for the local region and alters the conformation of heme distal His64. As a consequence, the fluoride ion interacts differently with the distal His64, as well as the water molecules in the heme distal pocket, compared to that in WT Mb. Kinetic study of fluoride binding to the protein further revealed that the micro-environmental changes in the heme active site enhance the rate of fluoride binding, as well as its dissociation from the heme iron. These observations indicate that the disulfide bond of Cys21–Cys66 in V21C/V66C Mb double mutant plays crucial roles in regulating both protein stability and ligand binding property, resembling the roles of Cys38–Cys83 in native Cgb. Therefore, the combination of both theoretical and experimental studies provides valuable information for understanding the structure and function of heme proteins, as regulated by a disulfide bond. Moreover, with the fine-tuning role of an intramolecular disulfide bond, this study is able to guide the rational design of artificial proteins with tunable functions in the future.

## 4. Materials and Methods

### 4.1. Materials and Reagents

Wild-type sperm whale Mb was expressed using the Mb gene of pMbt7–7 and purified using the procedure described previously [28]. V21C/V66C Mb mutant was prepared as reported in our previous study [33]. KH_2_PO_4_, NaF, and other chemicals were commercial products and of analytic grade. Deionized water was used throughout the experiments.

### 4.2. Molecular Dynamics Studies

The initial structure of V21C/V66C Mb was constructed based on the X–ray crystal structure of WT Mb (PDB code 1JP6 [31]) using program VMD 1.9. The heme axial water molecule in the X–ray structure was replaced by a fluoride ion for simulation of the fluoride–Mb complex. A patch of disulfide bond was applied to Cys21 and Cys66 for simulation of V21C/V66C Mb with an intramolecular disulfide bond. The protein was then solvated in a cubic box of TIP3 water, which extended 10 Å away from any given protein atom. Counter ions (Na^+^ and Cl¯) were further added to obtain the physiological ionic strength of 0.15 M by using the autoionize plug-in of VMD 1.9 [45]. The resulting system was minimized with NAMD2.9 (Nanoscale Molecular Dynamics) [46] using 50,000 minimization steps with conjugate gradient method at 0 K, and equilibrated for 40,000,000 molecular dynamics steps (1 fs per step, 40 ns in total) at 300 K, then further minimized for 50,000 steps at 0 K. The trajectory data were saved every 10,000 steps, and control simulation of fluoride binding to WT Mb was performed under the same conditions. Visualization and data analysis were done with VMD 1.9.

### 4.3. UV–Vis Kinetic Studies

UV–Vis spectra were recorded in 100 mM KH_2_PO_4_ (pH 7.0) on a Hewlett-Packard 8453 diode array spectrometer. Protein concentration was determined with an extinction coefficient of ε_409_ = 157 mM^−1^·cm^−1^ for WT Mb [47]; ε_409_ = 150 ± 5 mM^−1^·cm^−1^ for F46C/M55C Mb [33]. Fluoride binding to the heme center of WT Mb and F46C/M55C Mb was carried out with a SF-61DX2 Hi-Tech KinetAsyst^TM^ dual mixing stopped-flow spectrophotometer. The binding kinetics was measured by mixing the protein (10 μM in 100 mM KH_2_PO_4_ buffer, pH 7.0) in one syringe with increasing concentrations of NaF (0.1–0.4 M) in the second syringe, with an equal volume of solutions. The observed rate constant (*k*_obs_) was obtained by fitting the change of the protein Soret band to the mono-exponential decay equation. The association rate constant, *k*_a_ (*k*_on_), and the dissociation rate constant, *k*_d_ (*k*_off_), were determined from a plot of *k*_obs_ versus the fluoride concentration (i.e., *k*_obs_ = *k*_on_ [F^¯^] + *k*_off_), where the slope and intercept correspond to *k*_on_ and *k*_off_, respectively.

## Figures and Tables

**Table 1:** Comparison of the kinetic parameters of fluoride binding to WT Mb and the double mutant of V21C/V66C Mb with an intramolecular disulfide bond.

Proteins	*k*_on_ (M^−1^ s ^−1^)	*k*_off_ (s ^−1^)	*k*_on_/*k*_off_ (M^−1^ )
WT Mb	5.14 ± 0.15	0.13 ± 0.01	39.5
V21C/V66C Mb	12.48 ± 0.26	0.27 ± 0.04	46.2
